# Novel Genetic Diversity and Geographic Structures of *Aspergillus fumigatus* (Order Eurotiales, Family Aspergillaceae) in the Karst Regions of Guizhou, China

**DOI:** 10.3390/microorganisms14010237

**Published:** 2026-01-20

**Authors:** Duanyong Zhou, Yixian Liu, Qifeng Zhang, Ying Zhang, Jianping Xu

**Affiliations:** 1 Key Laboratory of Agricultural Microbial Resources Development and Utilization of Qianxinan Prefecture, Minzu Normal University of Xingyi, Xingyi 562400, China; zhouduanyong@xynun.edu.cn (D.Z.); zhangqifeng31@163.com (Q.Z.); 2 Research Centre of Plant Protection, Yunnan Institute of Tropical Crops, Jinghong 666101, China; liuyixian20251209@163.com; 3 State Key Laboratory for Conservation and Utilization of Bio-Resources in Yunnan, Yunnan University, Kunming 650500, China; 4 Department of Biology, McMaster University, Hamilton, ON L8S 4K1, Canada

**Keywords:** genetic diversity, genetic differentiation, population structure, phylogenetic analysis, azole susceptibility

## Abstract

*Aspergillus fumigatus* is the primary pathogen causing aspergillosis. Recent molecular population genetic studies have demonstrated that *A. fumigatus* exhibits high local genetic diversity, with evidence for limited differentiation among geographic populations. However, research on the impacts of geomorphological factors on shaping the population genetic diversity patterns of this species remains scarce. In this study, large-scale sampling and in-depth population genetic analysis were performed on soil-derived *A. fumigatus* from Guizhou Province, a representative karst landscape in southern China. This area is dominated by plateaus and mountains (accounting for 92.5% of the total area) and represents a classic example of conical karst landscapes. A total of 206 *A. fumigatus* strains were isolated from 9 sampling sites across Guizhou. Genetic diversity, genetic differentiation, and population structure of these strains were analyzed based on short tandem repeats (STRs) at 9 loci. The results revealed that *A. fumigatus* in the karst region of Guizhou harbors abundant novel alleles and genotypes, with high genetic diversity. Gene flow among geographical populations was infrequent, and significant genetic differentiation was detected between 30 of the 36 pairs of geographical populations where mountain ranges played a very important role, with the overall regional genetic differentiation reaching PhiPT = 0.061 (*p* = 0.001). Furthermore, the Guizhou populations showed significant differences from those reported in other regions worldwide. Surprisingly, only one of the 206 (0.49%) *A. fumigatus* isolates from this region exhibited resistance to the two medical triazoles commonly used for treating aspergillosis, and this resistance frequency was far lower than those reported in previous studies from other regions. We discuss the implications of our results for evolution and environmental antifungal resistance management in this important human fungal pathogen.

## 1. Introduction

*Aspergillus fumigatus* is a common saprophytic fungus widely distributed across diverse ecological niches worldwide. It is frequently found in soil, air, water, and the vicinity of plant roots, as well as in organic-matter-rich environments like compost and decaying vegetation [1,2,3,4]. As the most prevalent airborne opportunistic fungal pathogen in humans, *A. fumigatus* poses the gravest threat to immunocompromised individuals [5,6,7]. It is estimated that invasive aspergillosis affects nearly 10% of at-risk patients globally each year, with a case-fatality rate that can reach as high as 88% in specific high-risk cohorts [8,9]. Triazoles, including itraconazole (ITR), voriconazole (VOR), posaconazole (POS), and isavuconazole (ISA), are the first-line agents for treating invasive *A. fumigatus* infections [4,10]. However, since the first case of itraconazole-resistant *A. fumigatus* was reported in the United States in 1997 [11], azole-resistant *A. fumigatus* (ARAF) strains have been identified in most countries and are associated with a grave prognosis, as illustrated by a case series where invasive aspergillosis (IA) linked to these strains had a 100% 90-day mortality rate (n = 10) [12,13]. The emergence of ARAF constitutes a major challenge to human health and environmental safety [14,15,16].

Evidence increasingly points to a concerning global trend of rising ARAF infections, with resistant strains now being detected even in triazole-naïve patients [17,18,19]. These results indicate that many ARAF strains in triazole-naïve patients may be directly acquired from environmental populations. Consequently, understanding the prevalence and spread of triazole resistance in environmental *A. fumigatus* populations is thus essential for preventing ARAF aspergillosis outbreaks.

Guizhou, located in southwest China, is known as the “Karst Province”—with 73.6% of its land area composed of karst landforms and 92.5% covered by mountains and hills [20,21,22]. Due to the extremely fragile ecological environment and the existence of numerous underground karst caves, the difficulty and cost of road construction in karst areas are far higher than in other regions. This kept Guizhou Province in an almost closed state through most of its history [21]. In recent decades, there has been considerable research interest in the genetic diversity and structural patterns of Guizhou’s plants and animals, encompassing key species like wild tea [23], cultivated rice [24], indigenous chicken [25], and *Dendrothrips minowai*, commonly known as stick tea thrip, a significant pest in tea plantations across China, Japan, and Korea [26]. A recent study has shown that ascomycetes in this region are highly diverse [27]. However, the links between genetic structure, geological factors, and the evolutionary histories of fungi remain relatively poorly understood in Guizhou.

In this study, we sampled extensively and investigated the population structure of *A. fumigatus* from vegetable gardens at nine distinct geographic locations in Guizhou Province. A panel of nine short tandem repeat (STR) markers, which have been widely used for genotyping strains of *A. fumigatus* [28], were used to determine the genotypes of our strains. In addition, we investigated the susceptibilities of the strains to two common medical triazoles used for treating aspergillosis, itraconazole and voriconazole. For ARAF strains, we analyzed the DNA sequence at the azole target gene *cyp51A*. The obtained genotype information was compared with that from other parts of the world. We aimed to (i) reveal the genetic structure of *A. fumigatus* from the karst areas in Southwest China, (ii) explore the relative roles of geographical barriers and landscape features in shaping the population genetic diversities of this species, and (iii) investigate the prevalence of triazole-resistant strains and their potential mutations associated with azole resistance. Here, we hypothesize that the mountainous environment and inconvenient transportation in Guizhou Province limit the dispersal of *A. fumigatus*, thereby affecting its genetic structure. Furthermore, our previous studies showed high-frequency ARAF in greenhouses and outdoor environments, including agriculture fields such as vegetable gardens in Yunnan, a neighboring province west of Guizhou [29,30]. Given the high dispersal ability of *A. fumigatus* and the selective advantage ARAF strains have in the presence of triazole drugs, we hypothesize that ARAF will be similarly frequently found in Guizhou Province as in Yunnan Province.

## 2. Materials and Methods

### 2.1. Soil Samples, A. fumigatus Isolation and Identification

**Figure 1:**
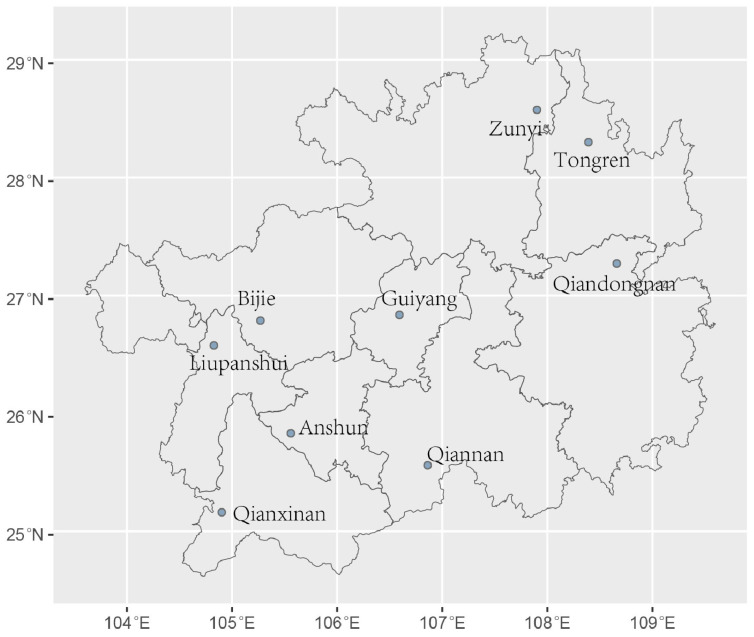
Detailed information of geographical distributions of *Aspergillus fumigatus* samples analyzed in this study.

A total of 900 soil samples were collected from vegetable gardens at nine distinct locations in Guizhou Province between 20 August and 1 September 2023. At each sampling site, 100 subsamples (approximately 10 g each) were obtained from the topsoil layer (0–5 cm depth) and immediately placed into sterile zip-lock plastic bags for preservation. At each site, individual soil samples were about 1 m apart from each other. Detailed information regarding the sampling locations is provided in Table S1 and Figure 1. Isolation of *A. fumigatus* was performed following the protocol described previously [17]. Initial and final identification of the strains was conducted according to the methods reported in our prior studies [29,30].

### 2.2. STR Genotypes and Population Genetic Analyses

All *A. fumigatus* isolates were genotyped using a panel of nine highly polymorphic short tandem repeat (STR) markers (STRAf 2A, 2B, 2C, 3A, 3B, 3C, 4A, 4B, 4C) to elucidate the genetic relationships among strains and populations collected from vegetable gardens at nine locations in Guizhou Province. The number of tandem repeats at each STR locus was determined following the protocol described previously [28]. Alleles at the nine STR loci were combined to generate a multilocus STR genotype for each *A. fumigatus* strain. Additionally, genotypic data of *A. fumigatus* isolates from other countries—previously reported and deposited in the STRAf database (http://afumid.shinyapps.io/afumID, accessed on 1 August 2025)—were extracted and compared with the isolates obtained in Guizhou Province in the present study.

For population genetic analyses, *A. fumigatus* isolates collected from each of the nine sampling sites were designated as members of a distinct local geographic population. GenAlEx v6.1 software was employed to calculate the levels of genetic diversity within and genetic differentiation among the nine geographic populations in Guizhou Province. In addition, the Guizhou population was compared with ones from other parts of the world [31]. Mantel test was performed to examine the correlation between genetic differentiation and geographical distances among the studied populations in Guizhou. Analysis of molecular variance (AMOVA) was used to estimate the relative contributions of geographic separation and different population subdivisions, as aforementioned, to the overall genetic variation. STRUCTURE v2.3.3 software was employed to infer the optimal number of genetic clusters (K) from two datasets: one consisting of the *A. fumigatus* isolates from Guizhou Province, and the other a combined dataset incorporating all 750 global samples from a prior study [32,33,34]. PCoA (Principal Coordinate Analysis) and DAPC (Discriminant Analysis of Principal Components) were used to investigate genetic relationships between *A. fumigatus* isolates. MSTs (Minimum Spanning Trees) and an STR marker-based phylogenetic tree (implemented in the R adegenet package version 2.1.0) were utilized to cluster genotypes in association with their geographic origins [35]. MEGA v6 was used for sequence alignment of the *cyp51A* gene [36]. To investigate the reproductive mode of natural *A. fumigatus* populations in Guizhou Province, three genetic indices—index of association (IA), rBarD, and proportion of phylogenetically compatible pairwise loci (PrC)—were calculated following the protocol described previously [37].

### 2.3. Susceptibility of A. fumigatus Isolates and cyp51A Gene Sequencing

Two clinical azole drugs (ITR and VOR) commonly used for the treatment of aspergillosis were used to test the susceptibility of *A. fumigatus* isolated in this study following the methods described in the CLSI M38-A3 [38] and our previous studies [29,30]. Two primer pairs, A7 (5′-TCATATGTTGCTCAGCGG-3′) and P450-A2 (5′-CTGTCTCACTTGGATGTG-3′) [39], were used for amplifying and sequencing the full-length *cyp51A* gene (encompassing coding and promoter regions) from ARAF strains isolated in this study.

## 3. Results

### 3.1. Genotyping of A. fumigatus Samples from Guizhou Province

In this study, 206 strains of *A. fumigatus* were isolated from 900 soil samples collected from vegetable gardens at nine locations in Guizhou Province, China (Figure 1, Table S1). Based on genotyping at the nine STR loci, a total of 212 alleles and 161 multilocus genotypes were identified. The number of alleles per STR locus ranged from 10 (STRAF4C, uh = 0.68) to 52 (STRAF3A, uh = 0.96), with an average of 24. Among the 212 alleles, 155 were shared by at least two geographical populations, and the remaining 57 were found only in one geographical population each. Private alleles were detected in every geographical population, with the Bijie population (n = 12) having the highest number of private alleles and the Liupanshui population (n = 1) the lowest. Among the 161 multilocus genotypes, 2 were shared by two geographical populations, and the remaining 159 were found only in one geographical population each (Table 1).

Genetic diversity analysis by population revealed the following results for the nine geographic populations of *A. fumigatus* in Guizhou Province: the number of effective alleles (Ne) ranged from 5.132 (Anshun population) to 7.426 (Bijie population), with a mean of 6.087; Shannon’s Information Index (I) varied from 1.679 (Anshun population) to 2.026 (Qiandongnan population), with an average of 1.849; Simpson’s Diversity Index (h) spanned 0.731 (Guiyang population) to 0.835 (Qiandongnan population), with a mean of 0.783; and Unbiased Diversity (uh) ranged from 0.769 (Guiyang population) to 0.881 (Qiandongnan population), with an average of 0.830 (Table 2).

### 3.2. Relationships Among Local Populations

**Figure 2:**
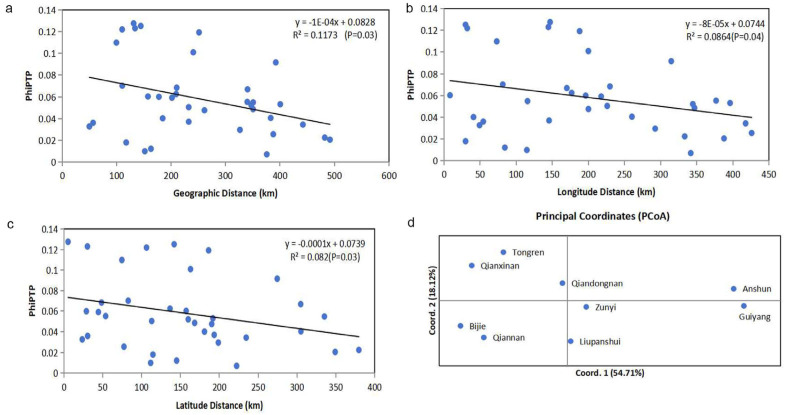
Results of Mantel test and PCoA. (**a**) Correlation analysis of genetic differentiation (PhiPTP) and geographical distance of *A. fumigatus* populations from Guizhou (*p* = 0.03). (**b**) Correlation analysis of genetic differentiation (PhiPTP) and longitude distance of *A. fumigatus* populations from Guizhou (*p* = 0.04). (**c**) Correlation analysis of genetic differentiation (PhiPTP) and latitude distance of *A. fumigatus* populations from Guizhou (*p* = 0.03). (**d**) Principal coordinate analysis of the mean population haploid genetic distance among 9 geographical populations in Guizhou.

**Figure 3:**
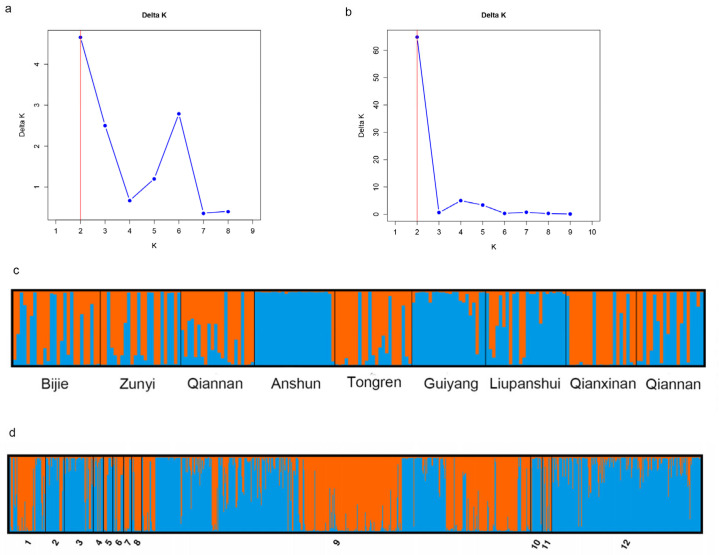
Genetic clustering results obtained from STRUCTURE analysis. Plot of K against delta K (**a**) and cluster assignments for isolates (**c**) for 9 geographic populations from Guizhou. Plot of K against delta K (**b**) and cluster assignments for isolates (**d**) based on data from 12 geographical populations from around the world, K = 2. 1, America; 2, South Asia; 3, East Asia; 4, Middle Asia; 5, Africa; 6, South Europe; 7, Middle Europe; 8, North Europe; 9, West Europe; 10, Oceanica; 11, unclear regions; 12, Guizhou, China.

Results of analysis of molecular variance (AMOVA) based on clone-corrected data showed that 94% of the total genetic variation originated from within individual geographical populations. While the level of genetic differentiation among the nine geographical populations was modest (6%), this difference was statistically significant (PhiPT = 0.061, *p* = 0.001) (Table S2). We additionally explored the degree of genetic differentiation between pairs of geographical populations. Our findings revealed that 30 of the 36 pairwise local populations showed statistically significant differentiation (*p* < 0.05). The greatest genetic differentiation was observed between Guiyang and Bijie populations (PhiPT = 0.128, *p* = 0.001), followed by that between Guiyang and Qiannan populations (PhiPT = 0.125, *p* = 0.001). The geographical populations from Qiannan and Bijie showed significant genetic differentiation from all other populations (*p* < 0.05) (Table 3). Interestingly, Mantel test showed that genetic differentiations among geographic populations were negatively correlated with geographical distance (R^2^ = 0.1173, *p* = 0.03) (Figure 2a), longitude distance (R^2^ = 0.0864, *p* = 0.04) (Figure 2b), and latitude distance (R^2^ = 0.082, *p* = 0.03) (Figure 2c). Multilocus analysis results revealed limited but unambiguous evidence for recombination among the nine STR loci in the total sample, which included all 206 *A. fumigatus* isolates from Guizhou (PrC = 0, *p* = 1; rBarD = 0.079, *p* < 0.001). STRUCTURE analysis showed that there were two genetic clusters (K = 2) of *A. fumigatus* from Guizhou (Figure 3a). However, these two genetic clusters were not widely or evenly distributed among the nine geographic populations. For example, isolates from Anshun and Guiyang populations mainly belonged to one cluster (blue), while isolates from populations in Qiannan, Tongren, and Qianxinan mainly belonged to the other cluster (orange) (Figure 3c). Principal coordinate analysis (PCoA) was performed based on the mean population haploid genetic distance. The first principal coordinate (PC1) contributed 54.71% of the total variance, and the second (PC2) accounted for 18.12%. Together, these two coordinates explained 72.83% of the total genetic variation. As shown in (Figure 2d), populations from Anshun and Guiyang were clearly distinct from the other geographic populations.

### 3.3. Relationship Between the Guizhou Population of A. fumigatus and Those in Other Global Regions

To further investigate the relationship between *A. fumigatus* samples from Guizhou and those from other regions around the world, we downloaded the information of 750 *A. fumigatus* samples from previous studies from the database (https://afumid.shinyapps.io/afumID/, accessed on 1 August 2025), including their sources and the genotype data at the same nine STR loci. Therefore, a total of 956 *A. fumigatus* strains were used for analysis in this section, including 206 strains from Guizhou and 750 strains from other regions worldwide. Based on their geographical origins, the 956 strains were divided into 12 geographical populations. Using the nine STR loci, we identified a total of 322 alleles, among which 197 alleles were shared between the Guizhou samples and other geographic populations. Of the remaining 125 alleles, 15 and 110 alleles were unique to the Guizhou population and the global populations, respectively (Table 4). Interestingly, all nine local populations were found to contain private alleles not found in the global population, with Guiyang containing the highest number, at 5 private alleles, while Liupanshui, Zunyi, and Tongren contained 1 private allele each (Table 4). A total of 838 multilocus genotypes were found from the 956 *A. fumigatus* strains. However, there was no shared multilocus genotype between Guizhou and other global geographic populations.

**Figure 4:**
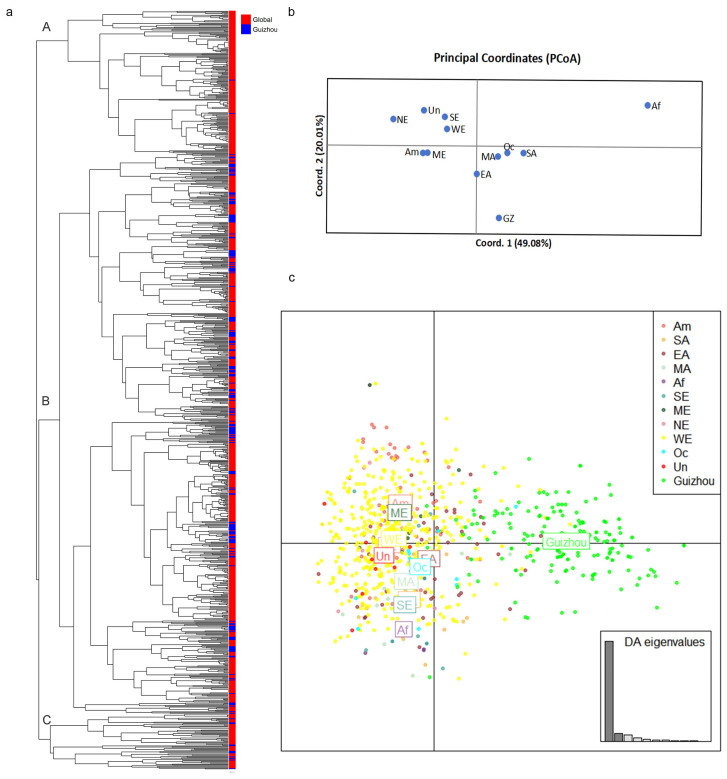
Phylogenetic analysis and results of PCoA and DAPC. (**a**) Phylogenetic analysis of isolates from 9 geographic populations in Guizhou (blue) and 11 geographic populations (Global) from outside of Guizhou (red). (**b**) Principal coordinate analysis of the mean population haploid genetic distance among 9 geographical populations in Guizhou and 11 geographic populations from outside of Guizhou. (**c**) DAPC analysis of 12 global geographical populations of *Aspergillus fumigatus*, AM, America; SA, South Asia; EA, East Asia; MA, Middle Asia; Af, Africa; SE, South Europe; ME, Middle Europe; NE, North Europe; WE, West Europe; Oc, Oceania; Un, unclear regions; GZ, Guizhou, China.

**Figure 5:**
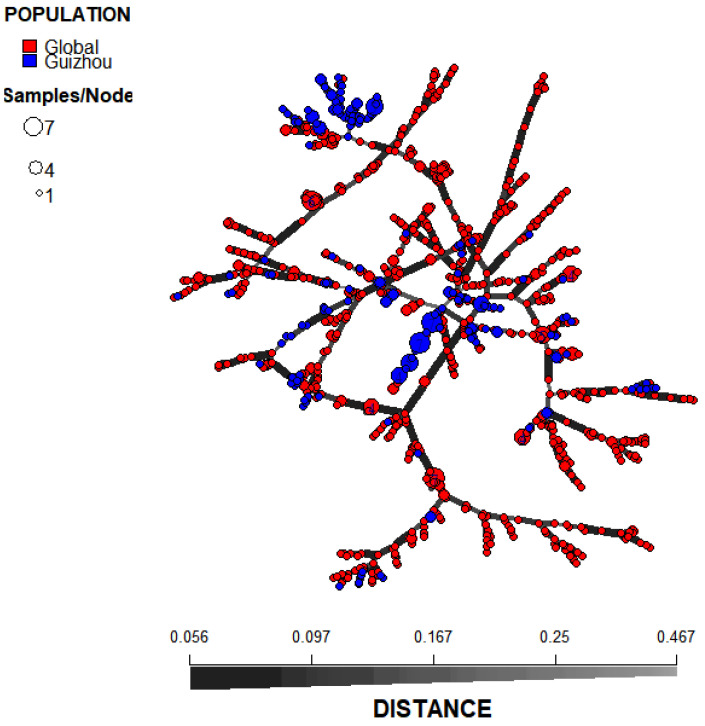
Minimum spanning tree (MST) showing the genotypic relationship among Global populations, including America, South Asia, East Asia, Middle Asia, Africa, South Europe, Middle Europe, North Europe, West Europe, Oceania, unclear regions and Guizhou, China.

Pairwise population analysis results revealed that among the 66 population pairs, 54 pairs exhibited statistically significant genetic differentiation (*p* < 0.05). Notably, the Guizhou population showed significant differentiation from all other geographic populations (*p* = 0.001). This finding was consistent with the results of the population structure analysis (Figure 3d). Specifically, the Northern Europe population displayed the highest degree of genetic differentiation from the Guizhou population (PhiPT = 0.114, *p* = 0.001), while the East Asian population that is geographically close to Guizhou showed the lowest degree of differentiation (PhiPT = 0.049, *p* = 0.001) (Table S3). Principal Coordinate Analysis (PCoA) results demonstrated that at a large geographical scale, *A. fumigatus* populations from Asia, Europe, and Africa each formed distinct genetic clusters. At a smaller geographical scale, the Guizhou *A. fumigatus* population was also distinguishable from those in East Asia, Central Asia, and South Asia (Figure 4b). Discriminant Analysis of Principal Components (DAPC) results demonstrated that *A. fumigatus* samples from Guizhou were primarily distributed on the right side of the coordinate plot, whereas the remaining global samples were predominantly localized on the left side (Figure 4c). The MSN results showed that the *A. fumigatus* strains from Guizhou belonged to multiple independent lineages (Figure 5). The results of phylogenetic analysis constructed based on Bruvo’s distance indicated that the strains sampled from Guizhou and global reference strains were clustered into three distinct groups (A, B, and C). Specifically, among the Guizhou isolates, 2 strains fell into group A, 192 strains were assigned to group B, and 12 strains were grouped into group C (Figure 4a). These findings indicated that the *A. fumigatus* population from Guizhou contains both unique and shared genetic characteristics with those from other global samples.

### 3.4. Prevalence of Azole Resistance and cyp51A Mutation

The antifungal susceptibility test results showed that among 206 *A. fumigatus* strains from Guizhou, only 1 strain isolated from Zunyi was resistant to both itraconazole and voriconazole, with a minimum inhibitory concentration (MIC) of ≥16.0 µg/mL for both antifungals. The remaining 205 strains were susceptible to both itraconazole and voriconazole. For the other strains, the MIC values of itraconazole ranged from 0.015 to 0.5 µg/mL, with an MIC50 of 0.063 µg/mL. The MIC values of voriconazole ranged from 0.031 to 0.25 µg/mL, with an MIC50 of 0.063 µg/mL. We amplified and obtained the *cyp51A* gene and promoter sequences of the one azole-resistant *A. fumigatus* strain using specific primers. The alignment results revealed that the one azole-resistant *A. fumigatus* strain belonged to the TR34/L98H mutation type.

## 4. Discussion

In this study, we isolated and identified 206 strains of *A. fumigatus* from soil samples collected from nine sites in Guizhou Province, China. Nine STR markers were employed to identify the genotypes of these isolates, as well as to analyze both the intra-population genetic diversity and inter-population relationships of the species. Our study found that the samples from the nine sites in Guizhou exhibited high levels of genetic diversity and genetic differentiation, with the Unbiased Diversity (uh) and PhiPT values being 0.830 and 0.061 (*p* = 0.001), respectively. Interestingly, among the nine sampling sites, only one ARAF strain was identified, in Zunyi. The azole resistance frequency of *A. fumigatus* strains isolated from nine vegetable gardens in Guizhou was 0.49% (1/206), far lower than previous reports from other provinces in China. A comparative analysis was further performed between the *A. fumigatus* strains isolated in this study and publicly available global strains. The results indicate that Guizhou-derived *A. fumigatus* exhibits both distinct characteristics and shared traits with isolates from other geographical regions.

### 4.1. Extensive Novel Genetic Diversity in Guizhou Province

Our previous studies showed extensive genetic uniqueness and high genetic diversity in *A. fumigatus* populations isolated from greenhouses, outdoor environments and the Three Parallel Rivers region across Yunnan, a neighboring province west of Guizhou [29,30,40]. In the present study, high levels of genetic diversity were also detected within and among nine distinct *A. fumigatus* populations in Guizhou Province. For instance, when examining the same nine STR loci, the number of private alleles and unique genotypes from the nine local populations in Guizhou Province was 57 and 159, respectively, accounting for 26.9% (57/212) of the total number of alleles and 98.8% (159/161) of the total number of genotypes, and all nine local populations had private alleles (Table 1). By comparing the results of these four studies, we found that both the proportions of private alleles and location-specific genotypes within local populations in Guizhou were the highest, followed by those from Yunnan’s greenhouses (19.2% and 97%, respectively) [30] and the Three Parallel Rivers region (19.1% and 98.8%, respectively) [40], while the population from Yunnan’s outdoor populations had the lowest (16.2% and 94.1%, respectively) [29]. The overall genetic diversity of samples from Guizhou (uh = 0.83) was slightly higher than that from Yunnan’s outdoor environments (uh = 0.827) [29] and the Three Parallel Rivers region (uh = 0.751) [40]. Additionally, comparison with the global AfumID database revealed that the Guizhou populations possessed 15 unique alleles and did not share any genotypes. Of these 15 private alleles, 3 were found at three of the nine sites, while the remaining 12 were each found at only one site (Table 4), suggesting the genetic uniqueness of each local population. These findings are consistent with previous studies on the genetic diversity of *A. fumigatus* in other regions worldwide, which all identified a large number of novel alleles and substantial genotypic diversity [17,41,42,43].

The presence of many private alleles and unique genotypes among the nine geographical populations of *A. fumigatus* in Guizhou might be attributed to the following reasons. First, evidence of sexual reproduction was found both within and between the nine geographical populations, which was conducive to maintaining the genetic diversity of *A. fumigatus* [41]. Second, evidence showed that gene flow existed among the *A. fumigatus* populations in Guizhou. Although this gene flow was limited between certain population pairs, it likely contributed to the genetic diversity of the local *A. fumigatus* populations [40]. Third, the high diversity might be associated with the generally mild climatic conditions in Guizhou, as well as the soil physicochemical properties under the karst landform. The high genetic diversity of *A. fumigatus* in Guizhou was consistent with the findings of previous studies on other plants and animals. For example, He S et al. conducted a study on *Rhododendron pudingense* in Guizhou Province and found that the species exhibited a high level of genetic diversity, with observed heterozygosity (Ho) and expected heterozygosity (He) of 0.45 and 0.77, respectively [44]. Liu Y et al. conducted a study on seven Chinese indigenous chicken populations in Guizhou Province and found that the genetic diversity of these Guizhou breeds was higher than that of commercial breeds, with Ho ranging from 0.315 to 0.346 and He ranging from 0.318 to 0.338 [45].

In our present study, the genetic diversity of *A*. *fumigatus* populations in most local populations was higher than that of other organisms. For example, the two geographic populations with the lowest unbiased allelic diversity in Guizhou were from Guiyang and Anshun, at 0.769 and 0.788 respectively. These two populations exhibited significant genetic differentiation from other geographical groups within Guizhou. These two sampling sites both had sandy soils. We hypothesize that nutrient deficiency in sandy soil may have restricted the growth and reproduction of *A. fumigatus*, thereby reducing population density, impeding gene flow, and accelerating genetic differentiation. In addition, both regions experience a subtropical humid monsoon climate with similar temperature and precipitation patterns. These shared ecological conditions may impose similar selection pressures on local fungal populations, leading to genetic convergence. Furthermore, Guiyang and Anshun are geographically relatively close to each other (~100 km). Such a short distance could have contributed to high gene flow and high genetic similarity between their *A. fumigatus* populations.

### 4.2. High Level of Genetic Differentiation Among the Nine Geographical Populations

In this study, 83.3% (30/36) of pairwise comparisons between nine vegetable garden populations revealed significant genetic differentiation (*p* < 0.05). Overall, about 6% of the total genetic variation was among the geographic populations. The proportion of genetic variation among populations relative to the total variation was higher than that from Yunnan’s outdoor environments (4%) [29], the Three Parallel Rivers region (4%) [40], and Yunnan’s greenhouses (2%) [30], and equally higher than that reported recently in clinical and environmental samples from Yunnan (5%) [3]. Interestingly, we also found that the genetic differentiation of samples from nine vegetable gardens in Guizhou (PhiPT = 0.061) was higher than that of those from Yunnan’s greenhouses (PhiPT = 0.019) [30], Yunnan’s outdoor environments (PhiPT = 0.044) [29], and the Three Parallel Rivers (TPR) region (PhiPT = 0.015) [40], as well as higher than that of the recently published clinical and environmental isolates from Yunnan (PhiPT = 0.045) [3]. Both PCoA and DAPC results showed that the strains from Guizhou were clustered differently from most other geographic populations from AfumID. In addition, phylogenetic tree analysis based on Bruvo’s distance revealed that *A. fumigatus* populations from Guizhou and global samples were clustered into three clades. Only 2 isolates belonged to Clade A, 12 isolates were clustered in Clade C, and the remaining isolates fell into Clade B. This result was inconsistent with previous findings that samples from Yunnan and global isolates formed two clades [29,30,40], indicating the uniqueness of Guizhou isolates.

Interestingly, we found that the observed genetic differentiations among *A. fumigatus* populations in Guizhou showed a significant negative correlation with geographic distance (Figure 2a), longitude distance (Figure 2b), and latitude distance (Figure 2c). The above findings are puzzling, and the following reasons could have contributed to some of the observations. First, Guizhou features a typical conical karst landscape with dense peak forests and several mountain ranges separated by deep valleys which could restrict the air-borne dispersion and diffusion of *A. fumigatus* conidia under natural conditions. Indeed, several pairs of geographic populations that are located relatively close to each other but showed high genetic differentiations were separated by high mountains, e.g., paired sites in Bijie vs. Guiyang; Liupanshui vs. Anshun; and Anshun vs. Qianxinan. On the other hand, though geographic relatively distant from each other, pairs of populations in the southern and southeastern parts of Guizhou such as Qixinan vs. Qiangnan; and Qiannan vs. Qiandongnan had no obvious large mountain barriers and those population pairs showed low genetic differentiation with each other. Second, historically, Guizhou has long been isolated and underdeveloped with extremely inconvenient transportation, resulting in restricted dispersals due to human activities. Consequently, the conidia of *A. fumigatus* were likely not dispersed in a geographic distance-dependent manner. Third, Guizhou Province is located in a low-latitude mountainous area with significant topographic relief, where the elevation difference between the eastern and western regions exceeds 2500 m. As the terrain gradually raised from east to west, various meteorological elements exhibited distinct variations, giving rise to diverse microenvironments. Such heterogeneous habitats may have facilitated the genetic diversification and adaptive evolution of *A. fumigatus* populations in this region. Similarly, in the study of *Rhododendron bailiense* in this region, a relatively high level of genetic differentiation among populations was observed, with a genetic differentiation coefficient (F_ST_) of 0.1907 [46]. However, at present, the exact reason(s) for the negative correlation between genetic differentiation and geographic distance is unknown. For example, there is no known migration corridor such as rivers or highway systems that specifically connect the least differentiated but geographically distant populations such as between Zunyi (in northeastern Guizhou) and Liupanshui (in western Guizhou) or between Qianxinan (in southwestern Guizhou) and Tongren (in northeastern Guizhou) (Figure 1). Furthermore, neither altitude nor types of vegetable crops were correlated with the observed genetic differences. Additional samples from these geographic regions are needed to critically assess the potential underlying causes for the observed negative correlations.

### 4.3. Low Level of Azole Resistance

In this study, we found that the incidence of azole resistance in *A. fumigatus* isolated from nine sites in Guizhou was only 0.49% (1/206), which was significantly lower than that in our previous studies conducted in neighboring Yunnan Province [29,30,40] and other parts of China [47]. At present, the origin of this ARAF is not known. However, Zunyi where this ARAF strain was isolated is a popular tourist area for people from across China. It is thus possible that a tourist could have brought this strain from outside of Guizhou to Zunyi. On a global scale, although a similarly low rate of triazole-resistance was reported in the Canadian soil populations of *A. fumigatus* [48], the currently reported azole resistance rates of *A. fumigatus* populations in most geographic regions are a lot higher, up to 50% outside of China [49,50,51] and up to 80% within China [29,30,40]. The relatively low incidence of azole resistance in *A. fumigatus* from Guizhou is likely attributed to the following factors: First, the local government has vigorously promoted green agriculture and strictly regulated the use of pesticides and fungicides. Second, influenced by traditional farming concepts, farmers in Guizhou generally apply little or no pesticides and fungicides (including triazoles) in agricultural production activities in their own vegetable gardens to safeguard personal health. Third, the high mountains, deep valleys, and Karst features in Guizhou fragment the landscape, making it difficult to operate large industrial-scale farms that are often associated with high pesticide and fungicide usages. As a result, the fungicide selection pressure in agriculture fields in Guizhou might be very low, thus reducing the opportunity for the emergence and spread of ARAF. Fourth, the low frequency of ARAF in Guizhou may also be associated with Guizhou’s temperature conditions: the average summer temperature in Guizhou is around 24 °C, and the annual average temperature is approximately 15 °C. Low temperatures restrict the growth and reproduction of plant pathogenic fungi, thereby reducing the occurrence of fungal diseases and subsequently lowering the frequency of fungicide application by farmers. Fifth, our research revealed that highly limited gene flow among many regions in Guizhou. Those barriers to gene flow could have constrained the spread of azole resistance genes and genotypes of *A. fumigatus* in Guizhou.

It has been reported in the literature that in the 1970s, demethylation inhibitor (DMI) fungicides began to be extensively used in agricultural production for controlling plant fungal diseases [52]. Approximately a decade later, the TR34/L98H mutation at the *cyp51A* gene was first reported in *A. fumigatus* in the Netherlands [53]. To date, it has been reported in many countries across all five continents worldwide [42,54,55,56,57]. In China, the TR34/L98H mutation in clinical *A. fumigatus* isolates was first reported in 2011 [58]. Subsequently, this mutation type has been identified in multiple provinces [30,59]. Based on existing literature reports, TR34/L98H is the most prevalent mutation type in ARAF strains detected in environmental samples across China [29,30,40,47,59]. To the best of our knowledge, this is the first report of the TR34/L98H mutation in environmental samples from the karst region of Guizhou, China. Regardless of the mechanisms, the low frequency of triazole resistance of *A. fumigatus* in Guizhou compared to other regions in China suggest that the approaches taken by Guizhou government and farmers represent a potentially excellent example from which to develop broader strategies to limit the origins and spread of triazole resistance in this fungal pathogen and to reduce its threat to public health [16,60].

## 5. Conclusions

This study analyzed the genotypes and triazole susceptibilities of 206 strains of the human fungal pathogen *A. fumigatus*. These strains were isolated from soil samples of private vegetable gardens of farmers at nine sites broadly distributed across Guizhou Province in southern China. Different from many previous studies of *A. fumigatus* from other parts of China and from outside of China, we found significant genetic differentiations between most pairs of local populations within Guizhou. In addition, though most alleles were shared between the Guizhou population and those from outside of Guizhou, private alleles were found in all nine local populations in Guizhou, and the Guizhou population was overall genetically significantly different from those outside of Guizhou. Furthermore, though one triazole-resistant strain was found, the frequency of ARAF is among the lowest reported so far in the global environmental populations of *A. fumigatus*. Our results suggest that the government policy and farming practices in Guizhou could be developed as a potential general approach to limit the emergence and spread of drug-resistant human fungal pathogens.

## Figures and Tables

**Table 1:** Detailed information of geographical distributions and STR alleles of *A. fumigatus* isolates from Guizhou Province, China.

Population	No. of Strains	No. of Genotypes	Microsatellite Loci and Number of Alleles (Private Alleles)									
			2A	2B	2C	3A	3B	3C	4A	4B	4C	Total
Guiyang	22	20	10	8 (1)	10	17 (6)	6	7	4	7 (1)	4	73 (8)
Zunyi	24	15	8	12 (1)	12 (2)	10 (2)	11 (2)	10 (2)	7 (1)	5	5	80 (10)
Qiannan	22	22	9	6	11	13 (1)	9	10 (2)	9 (2)	5	5 (1)	77 (6)
Anshun	24	17	7	6	9	15 (3)	6	7 (2)	5 (1)	8	6	69 (6)
Qiandongnan	20	19	10	9	9 (1)	16 (3)	10	10	6 (1)	9 (1)	6	85 (6)
Qianxinan	21	14	7	8	7	10 (3)	8	11 (1)	6	5 (1)	3	65 (5)
Liupanshui	24	17	8	7 (1)	10	15	12	11	4	6	5	78 (1)
Bijie	26	23	8	9 (1)	13 (1)	20 (3)	11 (1)	16 (5)	10 (1)	4	4	95 (12)
Tongren	23	16	6 (1)	8	10 (2)	11	9	13	8	6	4	75 (3)
Total	206	161	16 (1)	18 (4)	26 (6)	52 (21)	23 (3)	36 (12)	18 (6)	13 (3)	10 (1)	212 (57)

**Table 2:** Summary genetic diversities of *Aspergillus fumigatus* populations from Guizhou Province, China.

Sampling Site	Effective Alleles (Ne)	Shannon’s Information Index (I)	Diversity (h)	Unbiased Diversity (uh)
Bijie	7.426	1.982	0.789	0.825
Zunyi	6.543	1.963	0.822	0.88
Qiannan	5.426	1.807	0.775	0.813
Anshun	5.132	1.679	0.732	0.778
Tongren	5.984	1.88	0.806	0.86
Guiyang	5.695	1.711	0.731	0.769
Liupanshui	6.068	1.85	0.785	0.834
Qianxinan	5.414	1.741	0.771	0.83
Qiandongnan	7.099	2.026	0.835	0.881

**Table 3:** Pairwise differentiations among 9 geographical populations of *Aspergillus fumigatus* in Guizhou.

Bijie	Zunyi	Qiannan	Anshun	Tongren	Guiyang	Liupanshui	Qianxinan	Qiandongnan	
	0.009	0.001	0.001	0.002	0.001	0.013	0.003	0.001	Bijie
0.030		0.002	0.007	0.009	0.006	0.264	0.078	0.158	Zunyi
0.062	0.055		0.001	0.001	0.001	0.005	0.002	0.001	Qiannan
0.122	0.041	0.123		0.001	0.204	0.001	0.001	0.001	Anshun
0.049	0.036	0.067	0.092		0.001	0.003	0.110	0.101	Tongren
0.128	0.037	0.125	0.010	0.101		0.001	0.001	0.001	Guiyang
0.033	0.007	0.050	0.070	0.053	0.060		0.002	0.031	Liupanshui
0.040	0.022	0.059	0.110	0.021	0.119	0.060		0.015	Qianxinan
0.055	0.012	0.048	0.052	0.018	0.068	0.026	0.034		Qiandongnan

The PhiPT values are shown at the bottom left half of the table while the corresponding *p* values are shown at the top right half of the table.

**Table 4:** Summary allelic richness at the nine STR loci for population of *Aspergillus fumigatus* from Guizhou and comparison with those in the global population.

Locus	No. of Alleles in All 12 Populations	No. of Alleles in Guizhou	Private Alleles in Guizhou (Location, Frequency of Private Allele)
STRAF2A	19	16	None
STRAF2B	25	18	6 (Liupanshui, 0.005), 31 (Guiyang, 0.005)
STRAF2C	30	26	6 (Qiannan, Tongren, Qianxinan, 0.015), 7 (Bijie, Qiannan, Anshun, 0.049), 29 (Bijie, 0.005), 30 (Zunyi, 0.005)
STRAF3A	86	52	64 (Qianxinan, 0.005), 104 (Bijie, 0.005), 106 (Guiyang, 0.015), 107 (Guiyang, 0.005)
STRAF3B	33	23	None
STRAF3C	49	36	35 (Anshun, 0.005), 38 (Bijie, 0.005)
STRAF4A	26	18	3 (Qiannan, Guiyang, Qiandongnan, 0.02)
STRAF4B	25	13	18 (Qiandongnan, 0.005), 21 (Guiyang, 0.005)
STRAF4C	29	10	None
Total	322	212	15

## Data Availability

The original contributions presented in this study are included in the article/Supplementary Material. Further inquiries can be directed to the corresponding authors.

## Supplementary Materials

The following supporting information can be downloaded at: https://www.mdpi.com/article/10.3390/microorganisms14010237/s1, Table S1: Detailed information of the nine sampling sites in Guizhou; Table S2: Results of AMOVA among nine geographic populations of the *A. fumigatus* isolates from Guizhou; Table S3: Genetic differentiations between continental and regional geographical populations of *A. fumigatus*.

## Abbreviations

The following abbreviations are used in this manuscript:

STRs	Short Tandem Repeats
ARAF	azole-resistant *A. fumigatus*
ITR	itraconazole
VOR	voriconazole
MIC	minimum inhibitory concentration
