# Supplementary material for: Novel Genetic Diversity and Geographic Structures of Aspergillus fumigatus (Order Eurotiales, Family Aspergillaceae) in the Karst Regions of Guizhou, China
Source: Microorganisms. 2026 Jan 20;14(1):237. doi: 10.3390/microorganisms14010237 (PMC12843956; doi:10.3390/microorganisms14010237)
Supplement: Supplementary file 1 [file microorganisms-14-00237-s001.zip › Table S1. Datailed information of sampling sites in Guizhou.pdf]

Table S1. Detailed information of the nine sampling sites in Guizhou.

Sampling site	longitude	Latitude	Altitude (meters above sea level)	Types of vegetables grown
Guiyang	106.59	26.84	1227	lettuce
Zunyi	107.90	28.58	878	cabbage
Qiannan	106.86	25.56	427	pepper
Anshun	105.56	25.83	1339	cabbage
Qiandongnan	108.66	27.27	436	ginger
Qianxinan	104.90	25.16	1503	cabbage
Liupanshui	104.83	26.58	1833	cabbage
Bijie	105.27	26.79	1626	pepper
Tongren	108.39	28.30	501	cabbage
