# Supplementary material for: Novel Genetic Diversity and Geographic Structures of Aspergillus fumigatus (Order Eurotiales, Family Aspergillaceae) in the Karst Regions of Guizhou, China
Source: Microorganisms. 2026 Jan 20;14(1):237. doi: 10.3390/microorganisms14010237 (PMC12843956; doi:10.3390/microorganisms14010237)
Supplement: Supplementary file 1 [file microorganisms-14-00237-s001.zip › Table S2. AMOVA result among sites in Guizhou.pdf]

Table S2. Results of analysis of molecular variance (AMOVA) among nine geographic populations of *As[ergillus fumigatus* from Guizhou.

**Source**	**df**	**SS**	**MS**	**Est. Var.**	**%**	**Value**	**P**
Among Pops	8	65.00	8.13	0.24	6%	0.061	0.001
Within Pops	154	573.82	3.73	3.73	94%	0.061	0.001
Total	162	638.82		3.97	100%		
