# Supplementary material for: Novel Genetic Diversity and Geographic Structures of Aspergillus fumigatus (Order Eurotiales, Family Aspergillaceae) in the Karst Regions of Guizhou, China
Source: Microorganisms. 2026 Jan 20;14(1):237. doi: 10.3390/microorganisms14010237 (PMC12843956; doi:10.3390/microorganisms14010237)
Supplement: Supplementary file 1 [file microorganisms-14-00237-s001.zip › Table S3. Pairwise differentiations between A. fumigatus isolates from 12 geographical populations..pdf]

Table S3. Genetic differentiations between continental and regional geographical populations of *A. fumigatus*.

Am	SA	EA	MA	Af	SE	ME	NE	WE	Oc	Un	GZ	
	0.001	0.001	0.001	0.001	0.001	0.074	0.001	0.001	0.001	0.002	0.001	Am
0.048		0.083	0.391	0.012	0.059	0.007	0.002	0.001	0.036	0.018	0.001	SA
0.050	0.008		0.446	0.001	0.002	0.009	0.001	0.001	0.002	0.001	0.001	EA
0.057	0.001	0.000		0.017	0.033	0.041	0.005	0.015	0.002	0.034	0.001	MA
0.108	0.051	0.076	0.060		0.134	0.019	0.007	0.001	0.001	0.016	0.001	Af
0.073	0.032	0.055	0.045	0.035		0.055	0.113	0.007	0.003	0.095	0.001	SE
0.022	0.048	0.030	0.031	0.081	0.047		0.017	0.051	0.001	0.030	0.001	ME
0.057	0.069	0.064	0.063	0.098	0.025	0.049		0.029	0.001	0.196	0.001	NE
0.033	0.023	0.025	0.020	0.062	0.026	0.018	0.017		0.001	0.189	0.001	WE
0.079	0.024	0.036	0.046	0.095	0.085	0.078	0.112	0.048		0.003	0.001	Oc
0.053	0.038	0.049	0.029	0.070	0.029	0.048	0.014	0.007	0.088		0.001	Un
0.092	0.070	0.049	0.064	0.107	0.112	0.081	0.114	0.079	0.080	0.103		GZ

Note: PhiPT Values below diagonal. Probability, P (rand >= data) based on 999 permutations is shown above diagonal. AM,America; SA, South Asia; EA, East Asia; MA, middle Asia; Af, Africa; SE, south Europe; ME, middle Europe; NE, north Europe; WE, west Europe; Oc, Oceanica;
UN, unclear regions; GZ, Guizhou, China
